# The Phytochemical Indicaxanthin Synergistically Enhances Cisplatin-Induced Apoptosis in HeLa Cells via Oxidative Stress-Dependent p53/p21^waf1^ Axis

**DOI:** 10.3390/biom10070994

**Published:** 2020-07-02

**Authors:** Mario Allegra, Antonella D’Anneo, Anna Frazzitta, Ignazio Restivo, Maria Antonia Livrea, Alessandro Attanzio, Luisa Tesoriere

**Affiliations:** Dipartimento di Scienze e Tecnologie Biologiche, Chimiche e Farmaceutiche, Università di Palermo, 90123 Palermo, Italy; mario.allegra@unipa.it (M.A.); antonella.danneo@unipa.it (A.D.); anna.frazzitta@unipa.it (A.F.); ignazio.restivo@unipa.it (I.R.); maria.livrea@unipa.it (M.A.L.)

**Keywords:** combo-therapy, oxidative stress, phytochemicals, indicaxanthin, *Opuntia ficus indica* (L. Mill), cancer, cisplatin

## Abstract

Combining phytochemicals with chemotherapics is an emerging strategy to treat cancer to overcome drug toxicity and resistance with natural compounds. We assessed the effects of indicaxanthin (Ind), a pigment obtained from *Opuntia ficus-indica* (L. Mill) fruit, combined with cisplatin (CDDP) against cervical cancer cells (HeLa). Measured cell viability via Trypan blue assay; cell morphology via fluorescence microscopy; apoptosis, cell cycle, mitochondrial membrane potential (MMP) and cell redox balance via flow-cytometry; expression levels of apoptosis-related proteins via western blot. Cell viability assays and Chou-Talalay plot demonstrated that the combination of CDDP and Ind had synergistic cytotoxic effects. Combined treatment had significant effects (*p* < 0.05) on phosphatidylserine externalization, cell morphological changes, cell cycle arrest, fall in MMP, ROS production and GSH decay compared with the individual treatment groups. Bax, cytochrome c, p53 and p21^waf1^ were over-expressed, while Bcl-2 was downregulated. Pre-treatment with N-acetyl-l-cysteine abolished the observed synergistic effects. We also demonstrated potentiation of CDDP anticancer activity by nutritionally relevant concentrations of Ind. Oxidative stress-dependent mitochondrial cell death is the basis of the chemosensitizing effect of Ind combined with CDDP against HeLa cancer cells. ROS act as upstream signaling molecules to initiate apoptosis via p53/p21^waf1^ axis. Ind can be a phytochemical of interest in combo-therapy.

## 1. Introduction

Cisplatin (*cis*-diamminedichloro platinum(II), CDDP) is a first-generation platinum complex chemotherapeutic drug extensively employed in the treatment of many human solid tumors such as cervical cancer [1]. Its mode of action has been linked to the ability to damage DNA through crosslinking with the purine bases. When the DNA damage is beyond repair, the cells undergo a programmed cell death [2]. In addition, other mechanisms, such as the production of reactive oxygen species (ROS), are involved in the drug toxicity. CDDP-induced oxidative stress primarily targets mitochondrion [3]. This results in loss of protein sulfhydryl groups, inhibition of calcium uptake and fall of mitochondrial membrane potential (MMP) [3]. In spite of its effectiveness, its severe adverse effects, including renal and hepatic dysfunction, anemia and leucopenia [4] as well as the generation of chemo-resistance, limit the clinical use of CDDP [5].

The drug combination therapy is considered an effective strategy in cancer treatment to overcome chemotherapy toxicity because of lower doses required. Recently there has been a growing interest in the use of natural chemo-preventive agents such as phytochemicals that, acting in synergy with commercial drugs, bring about an enhanced effect [6,7,8,9,10,11]. A large number of these compounds are commonly included in the human diet, bioavailable and, more importantly, do not exert toxic effects at the absorbed amounts.

**Figure 1:**
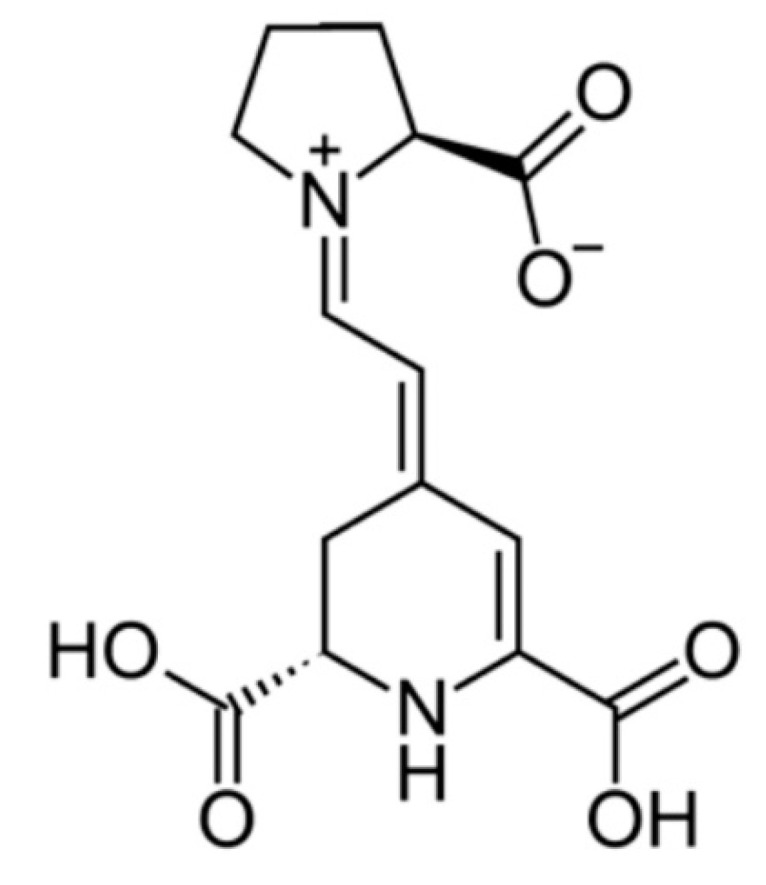
Molecular structure of indicaxanthin.

Indicaxanthin ((2*S*)-2,3-dihydro-4-[2-[(2*S*)-2α-carboxypyrrolidin-1yl]ethenyl]pyridine-2α,6-dicarboxylic acid, Ind), the yellow pigment characterizing the edible fruit of the cactus *Opuntia ficus-indica* (L. Mill), is a water-soluble alkaloid belonging to the class of betalain phytochemicals (Figure 1). Extensive research over the years has demonstrated that Ind has a variety of biological properties and exerts pharmacological antioxidant [12] and anti-inflammatory [13,14] effects. Most recently, its chemopreventive and antitumor activity in vitro and in animals has also been demonstrated [15,16,17]. In the human Caco2 colon cancer cell line, anti-proliferative and pro-apoptotic effects of Ind are associated with re-activation of the expression of the onco-suppressor p16 INK4a through the induction of the demethylation of its promoter region [15]. Ind inhibits the growth of other colorectal cancer cell lines, such as LOVO1, HCT116, and DLD1 cells and influences their DNA methylation status by affecting expression and activity of DNA methyltransferase enzyme [16]. In A375 human melanoma cells, Ind is able to induce apoptosis by inhibiting NF-κB activation and regulating the expression of NF-κB-dependent anti-apoptotic proteins [17]. Finally, in a xenograft model of cutaneous melanoma, the phytochemical orally administered to mice impairs tumor progression and reduces plasma levels of melanoma-associated chemokines [17]. On the other hand, interestingly, Ind, until 200–250 µM is not able to affect viability of non-cancer cells such as normal human epidermal melanocytes [17] or intestinal normal-like differentiated Caco-2 cells [15].

Based on this knowledge, the present study has assessed the effects of a combination of Ind and CDDP on growth, cell cycle progression and apoptosis in HeLa cells and researched molecular mechanisms involved in the observed synergism. Moreover, as Ind is a highly bioavailable phytochemical in humans [18], potentiation of anticancer activity of CDDP toward HeLa cells by nutritionally relevant concentrations of Ind has also been investigated.

## 2. Materials and Methods

All materials and reagents were acquired from Sigma-Aldrich Chemical Co (St. Louis, MO, USA), unless otherwise specified. Solvents were of HPLC grade. We purchased HeLa (cervical cancer cells) from the American Type Culture Collection (Rockville, MD, USA). Cells were cultured in Dulbecco’s modified Eagle’s medium (DMEM) supplemented with L-glutamine (2 mM), 10% fetal bovine serum (FBS), penicillin (100 U/mL), streptomycin (100 µg/mL) and gentamicin (5 μg/mL). Cells were maintained in log phase by seeding in flasks twice a week at a density of 5.0 × 10^4^ cells/cm^2^ in humidified 5% CO_2_ atmosphere, at 37 °C.

Ind was isolated from *Opuntia ficus indica* (L. Mill) fruit pulp (yellow cultivar) and purity (97%) of the pigment was assessed by HPLC according to a previously described method [17].

### 2.1. Cytotoxicity Assay In Vitro

The effect of CDDP and Ind on the cell viability was measured by trypan blue exclusion test [19]. Briefly, HeLa cells were seeded at 5.0 × 10^4^ cells/cm^2^ in 96-well plates containing 200 03BCL DMEM and allowed to adhere through overnight incubation. Then, cells in subconfluence were washed with fresh medium and incubated for 24 h with various concentrations of CDDP (from 1 to 100 μM) or Ind (from 1 to 200 μM) in DMEM. Before use, Ind (40 mM) in phosphate-buffered saline solution, pH 7.4, (PBS) was filter-sterilized (0.2 μm filters, Millipore, Milan, Italy). CDDP was dissolved in dimethyl sulfoxide (DMSO) and then diluted in culture medium so that the effective DMSO concentration did not exceed 0.1%. No differences were found between cells treated with 0.1% DMSO and untreated cells in terms of cell number and viability. After treatment, media were aspirated carefully and the plate gently washed with PBS. Then, cells attached to the culture plastic were collected after 0.25% trypsin-EDTA treatment and mixed with an equal volume of 0.4% trypan blue. Unstained (viable) and stained (nonviable) cells were separately counted in a hemocytometer chamber (Neubauer’s chamber) under a light microscope (Carl Zeiss, Oberkochen, Germany). Cell viability was expressed as a percentage of total number of viable cell divided by total number of cells. IC_50_ value was defined as the molar concentration of the compound that inhibits 50% cell viability and calculated by the dose-response inhibition model in GraphPad Prism 5.02 (GraphPad Software, San Diego, CA, USA).

### 2.2. Estimation of Combination Index to Determine Interaction of CDDP with Ind

The Chou and Talalay method was used to investigate the combined effect of CDDP and Ind [20]. HeLa cells were treated with a range of concentrations of CDDP in combination with Ind at a fixed concentration ratio (1: 6), chosen on the IC_50_ values of the compounds when used alone. The concentration ranges were: Cis: 5–25 µM; Ind: 30–150 µM. After 24 h, cell growth was examined using Trypan blue method as above reported. In selected experiments, pre-treatment with N-acetyl-L-cysteine (NAC, 2 mM) was done for 1 h before adding CDDP combined with Ind. The Combination Index (CI) was calculated using CompuSyn software (ComboSyn, Paramus, NJ, USA) to define the type of effect. CI is calculated for every fraction affected (*f_a_*) value, where *f_a_* is defined as percentage inhibition/100. CI > 1, CI < 1 and CI = 1 indicate antagonism, synergism and additive effect respectively. We used the same software to calculate the dose reduction index (DRI). This is defined as the comparison of the dose of each drug alone with the degree of dose reduction of the combined drugs for the same degree of effect.

### 2.3. Flow Cytometry

#### 2.3.1. Measurement of Phosphatidylserine Exposure

Cell double staining with annexin V/propidium iodide (PI) was used to detect externalization of phosphatidylserine to the membrane surface by flow cytometry. Tumor cells were seeded in triplicate in 24-wells culture plates at a density of 5.0 × 10^4^ cells/cm^2^. Cells were incubated overnight and washed with fresh medium. Then, individual compounds or in combination were added. After 24 h incubation, cells were harvested by trypsinization, adjusted at 1.0 × 10^6^ cells/mL with buffer according to the manufacturer’s instructions (eBioscience, San Diego, CA, USA) and submitted to double staining with annexin V/PI as previously described [21]. We analyzed samples of at least 1.0 × 10^4^ cells by fluorescence-activated cell sorting (FACS) using Epics XL™ flow cytometer (Expo32 software, Beckman Coulter, Fullerton, CA, USA).

#### 2.3.2. Cell Cycle Analysis

Aliquots of 1.0 × 10^6^ cells were harvested by centrifugation, washed with PBS and incubated for 30 min, at room temperature in the dark in a PBS solution containing Triton X100 (0.1%, *v*/*v*), 20 μg/mL PI and 200 μg/mL RNase. Then samples were immediately subjected to FACS analysis. At least 1 × 10^4^ cells were analyzed for each sample.

#### 2.3.3. Measurement of Mitochondrial Transmembrane Potential (MMP)

The cationic lipophilic dye 3,3′-dihexyloxacarbocyanine iodide (DiOC6) (Molecular Probes, Inc., Life Technologies Italia, Monza, Italy) was used to assess the MMP. DiOC6 accumulates in the mitochondrial matrix and a reduction in the DiOC6-induced fluorescence intensity indicates a decrease of mitochondrial membrane potential (ΔΨm). Cells were treated for 24 h, then incubated with DiOC6 at a 40 nmol/L final concentration, for 15 min at 37 °C. Cells were collected by centrifugation, washed with PBS and suspended in 500 μL PBS. Then samples of at least 1.0 × 10^4^ cells were subjected to FACS analysis.

#### 2.3.4. Intracellular Reactive Oxygen Species (ROS) and Glutathione (GSH)

Fluorescent probes 2′,7′-dichlorofluorescin diacetate (DCFDA) or 5-chloromethylfluorescein diacetate (CMFDA), were used to cytofluorimetrically measure intracellular levels of ROS and GSH, respectively [22]. Thirty minutes before ending the cell treatment, DCFDA or CMFDA at 10 μM and 1 μM final concentration, respectively, were added to the medium. Then the cells were washed with PBS, resuspended in the same buffer and analyzed.

### 2.4. Fluorescence Microscopy

After 24 h treatment, the cells were rinsed with PBS and stained with acridine orange and ethidium bromide (AO/EB) fluorescent dyes in Hank’s buffer, as described previously [23]. The cells were examined by fluorescence microscopy (Leica Microsystems, Wetzlar, Germany). Intact cells appeared green, with uncompromised nuclei. Chromatin condensation and nuclear fragmentation in early apoptotic cells caused them to be stained green with bright green dots. Lost membrane integrity and condensed and fragmented orange chromatin was displayed in late apoptotic cells. Condensed chromatin was not evident in necrotic cells nuclear morphology although also stained orange. Images were taken with an inverted fluorescence microscope equipped by a CCD camera (Leica Microsystems, Wetzlar, Germany). Rhodamine and fluorescein isothiocyanate (FITC) filters were used for red and green fluorescent cells, respectively. Micrographs reported in results section are merged after combining red and green channels by Leica Q Fluo software (Leica Microsystems, Wetzlar, Germany).

### 2.5. Western Blotting

After treatment, cold PBS was used to rinse the cells. Cells were suspended in cell lysis buffer [0.5% sodium deoxycholate, 150 mM NaCl, 0.1% SDS, 1% NP-40, 50mM Tris-HCl (pH 7.4), 20mM NaF, 50 mM glycerophosphate, 20 mM EGTA, 0.5 mM PMSF, 1 mM DTT, and 1 mM Na_3_VO_4_] containing phosphatase- and protease inhibitors and sonicated (3 cycles, each for 10 sec in a Soniprep 150, Cellai Srl, Milano, Italia). After vigorously vortexing, samples were centrifuged at 12,000 rpm at 4 °C for 30 min and supernatants were collected and stored at −80 °C. Bradford reagent was used to determine the concentration of proteins. Equal amounts of protein samples (40 μg/lane), were separated on 10% gel by discontinuous SDS-PAGE and then electrotransferred to a polyvinylidiene difluoride membrane (Millipore, Merck, Darmstadt, Germany). The immunoblot was incubated overnight at 4 °C with blocking solution (5% skim milk), followed by incubation with a 1: 200 dilution of anti-Bax monoclonal antibody (clone YTH6A7, Cat No. SC-80658, Santa Cruz Biotechnology, Santa Cruz, CA, USA), anti-Bcl-2 (C-2, Cat No. SC-7382, Santa Cruz Biotechnology), anti-cytochrome c (A-8, Cat No. SC-13156, Santa Cruz Biotechnology), anti-p53 (DO-1, Cat No SC-126, Santa Cruz Biotechnology) or anti-p21^waf1^ (F-5, Cat No SC-6246 Santa Cruz Biotechnology) for 1 h at room temperature. Blots were washed two times with Tris-buffered saline/Tween 20 (TBST) and incubated with a 1: 2000 dilution of horseradish peroxidase (HRP)-conjugated anti-IgG antibody (Dako Denmark, Glostrup, Denmark) for 1 h at room temperature. After washing with TBST, blots were developed by enhanced chemiluminescence (Amersham Life Science, Arlington Heights, IL, USA). Quantity One Imaging Software (Bio-Rad Laboratories, Hercules, CA, USA) was used to analyze the optical density of the bands. Ponceau red staining and immunoblotting for β-tubulin (D-10, Cat No. SC-5274, Santa Cruz Biotechnology) were used to verify the correct protein loading. The results of the densitometric analysis were reported as arbitrary densitometric units normalized to β-tubulin.

### 2.6. Statistical Analysis

Results are given as means ± standard deviations (SD). Unless stated otherwise, three independent observations were performed for each experiment, thrice replicated. Statistical software (GraphPad Software) with a test for normality followed by one-way ANOVA and Tukey’s correction was used. Mean values without a common symbol were significantly different at *p* < 0.05.

## 3. Results

### 3.1. Growth-Inhibitory Effect of CDDP and Ind Alone and in Combination

**Figure 2:**
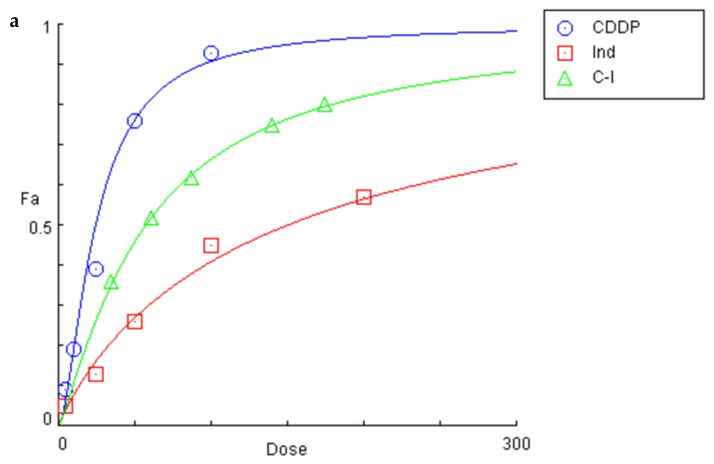

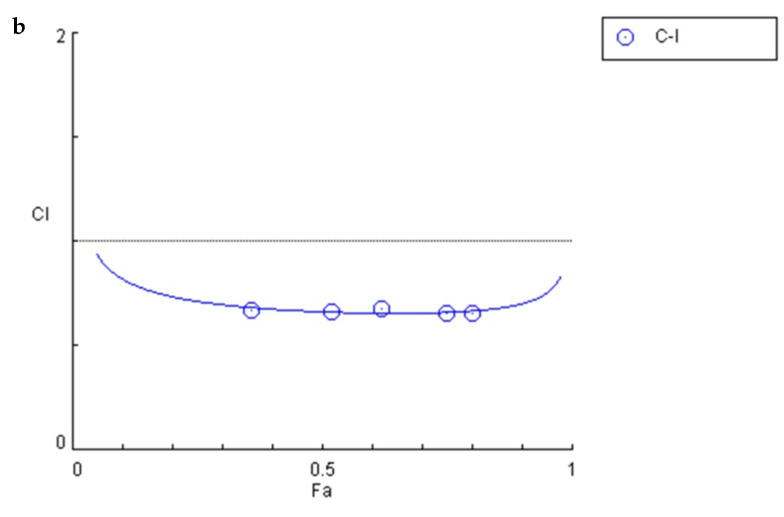
Effect on the viability of HeLa cells of CDDP (1–100 µM), Ind (1–200 µM), and CDDP combined with Ind (C-I) (5/30 µM; 7.5/45 µM; 12.5/75 µM; 20/120 µM, 25/150 µM) (**a**) and plot of the combination index vs. fraction of cells affected obtained using the median-effect analysis program (**b**).

The effects of CDDP, Ind and CDDP combined with Ind on the viability of HeLa cells were evaluated by the trypan-blue staining method (Figure 2a). After 24 h of treatment with the individual compounds, the calculated 50% inhibitory concentrations (IC_50_) of CDDP and Ind were 23.91 ± 1.3 µM and 149.55 ± 10.2 µM, respectively (*n* = 9). The inhibitory effect of CDDP/Ind co-treatment was studied using equipotent combinations of various doses of the two drugs according to the combination index theorem of Chou and Talalay based on the median-effect equation [20]. The combination index (CI) values for different inhibition fractions (*f_a_*) of growth of HeLa cells were calculated and are shown as a function of the effect level (Figure 2b). CI values were quite similar at each assayed dose and far below a value of 1.0, indicating synergism between the two drugs in a wide range of concentrations. The strongest synergy between the drugs (0.66) was calculated at 0.5 fractional inhibition of growth of HeLa, while the dose reduction index (DRI) values indicated that the effective CDDP dose in synergistic combination with Ind could be reduced to about 2 times and up to 3 times (Table 1). (C-I) (5/30 µM; 7.5/45 µM; 12.5/75 µM; 20/120 µM, 25/150 µM) (a) and plot of the combination index vs. fraction of cells affected obtained using the median-effect analysis program (b).

### 3.2. Induction of Apoptosis by CDDP and Ind Alone and in Combination

**Figure 3:**
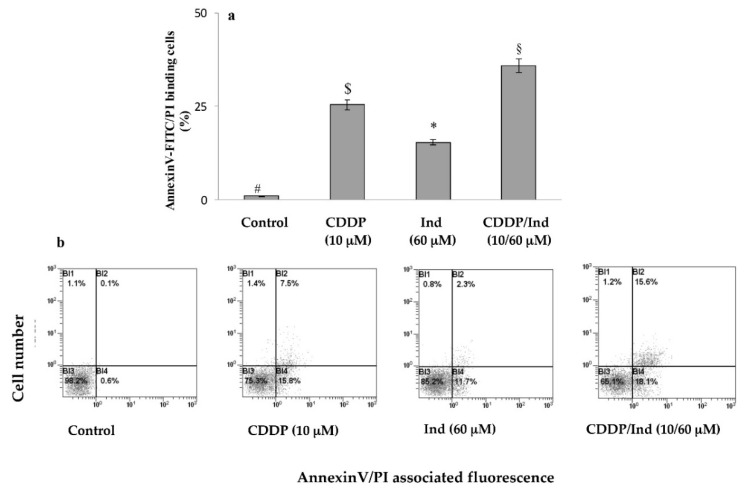
Apoptosis induced by CDDP, Ind and CDDP/Ind combination on HeLa cells. Cells were treated for 24 h as described in Methods. Percentage of AnnexinV/PI double stained-HeLa cells were determined by flow cytometer and compared to untreated cells (control). (**a**) Mean values ± SD of three separate experiments in triplicate. Bars without a common symbol are significantly different with *p* < 0.05 (one-way Anova associated with Tukey’s post hoc test); (**b**) Representative images: BI3, viable cells (AnnexinV−/PI−); BI4, cells in early apoptosis (AnnexinV+/PI−); BI2, cells in tardive apoptosis (AnnexinV+/PI+); BI1, necrotic cells (AnnexinV−/PI+).

To explore the mechanisms underlying the inhibitory effects of the two agents on HeLa cells proliferation, the fraction of apoptotic cells treated for 24 h with CDDP and Ind alone or in combination, at the ratio of their IC_50_ values (1: 6), was determined by flow cytometry using Annexin V-FITC/PI double staining. As shown in Figure 3 the percentage of apoptotic cells increased in both the CDDP (10 µM) and Ind (60 µM) groups compared with that of the control (25.4 ± 1.9 and 15.3 ± 2.8 vs 0.9 ± 0.1%). In addition, the CDDP/Ind combination treatment caused a more significant apoptosis than CDDP treatment alone (35.9 ± 2.6 vs 25.4 ± 1.9%).

**Figure 4:**
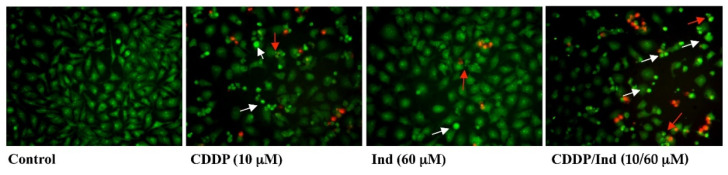
Cell apoptosis assessed by AO/EB double staining. Fluorescence micrographs representative of three separate experiments with similar results. White arrow: cells with chromatin condensation; red arrow: cells with membrane blebbing.

Characteristic morphological features of apoptotic HeLa cells treated with CDDP and Ind alone or in combination were assessed under a fluorescence microscope after double staining of the cells with AO/EB (Figure 4). Results displayed that apoptotic cells with condensed chromatin or affected cell permeability, which were green and orange-red, respectively, were more numerous in the CDDP/Ind groups than in CDDP or Ind groups.

### 3.3. Effect of CDDP and Ind Alone and in Combination on Cell Cycle Phase Distribution

**Figure 5:**
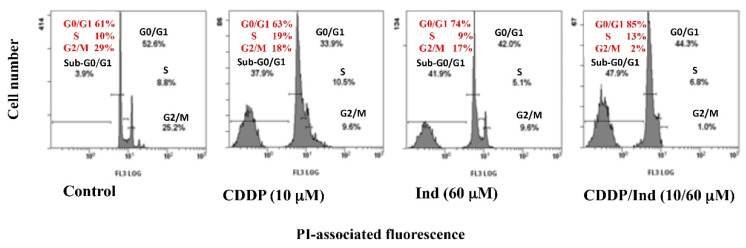
Effect of CDDP, Ind and CDPP/Ind combination on cell cycle distribution of HeLa cells. Cells were treated for 24 h as described in Methods. Then samples were submitted to flow cytometry analysis after propidium iodide (PI) staining and cell distribution compared to untreated cells (control). Each picture shows the percentage of viable cells in the different phases. Values are the mean ± SD of three separate experiments in triplicate. Representative images of three experiments with similar results.

Alterations in the cell cycle caused by a 24 h treatment of HeLa cells with CDDP and Ind were determined by labelling the DNA with propidium iodide and analyzing with flow cytometer. When compared to untreated cells (control), CDDP significantly reduced the number of cells in G2/M phase with a concomitant increased in S phase and appearance of a subG0/G1-cell population (Figure 5). On the contrary, treatment of the cells with Ind induced an accumulation of cells in phase G0/G1 with a reduction in G2/M, while percentage of cells in S phase appeared unchanged. Interestingly, when CDDP/Ind combination was used, the cell cycle distribution resulted significantly different (*p* < 0.05) from that observed. Indeed, the combined treatment caused a remarkable increase of cells in G0/G1 phase, an almost total absence of cells in G2/M phase and an increased percentage of sub G0/G1 cell population.

### 3.4. Effect of CDDP and Ind Alone and in Combination on Mitochondrial Membrane Potential (MMP)

**Figure 6:**
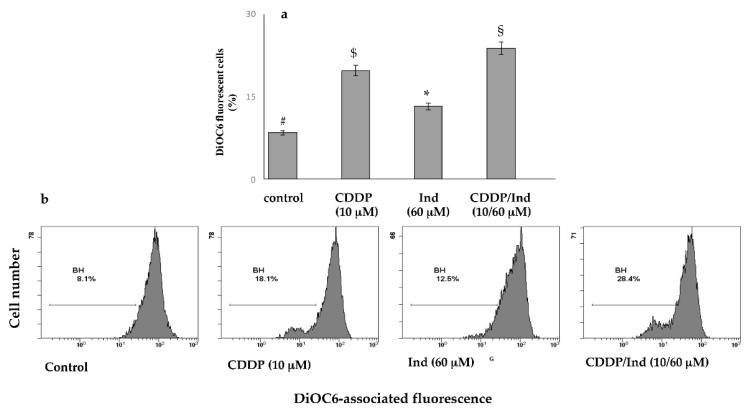
Depolarization of mitochondrial membrane potential induced by CDDP, Ind and CDDP/Ind combination on HeLa cells. Cells were treated for 24 h as described in Methods. Then percentage of DiOC6 stained-HeLa cells were determined by flow cytometer and compared to untreated cells (control). (**a**) Mean values ±SD of three separate experiments in triplicate. Bars without a common symbol are significantly different with *p* < 0.05 (one-way Anova associated with Tukey’s post hoc test); (**b**) Representative images.

Mitochondrial dysfunction is an early marker of mitochondria-mediated cell apoptosis. We measured the depolarization of MMP (ΔΨm) using DiOC6, a fluorescent mitochondria-specific and voltage-dependent dye. This showed that treatment for 24 h with 10 µM CDDP or 60 µM Ind alone, resulted in an increased of MMP depolarization compared to control groups (19.7 ± 1.6% and 13.2 ± 1.2%, respectively vs 8.4 ± 0.3%) (*p* < 0.05). However, with the combined use of CDDP/Ind, the ΔΨm increased to 23.8 ± 1.8% (Figure 6).

### 3.5. Effect of CDDP and Ind Alone and in Combination on Expression of Apoptosis-Related Proteins

**Figure 7:**
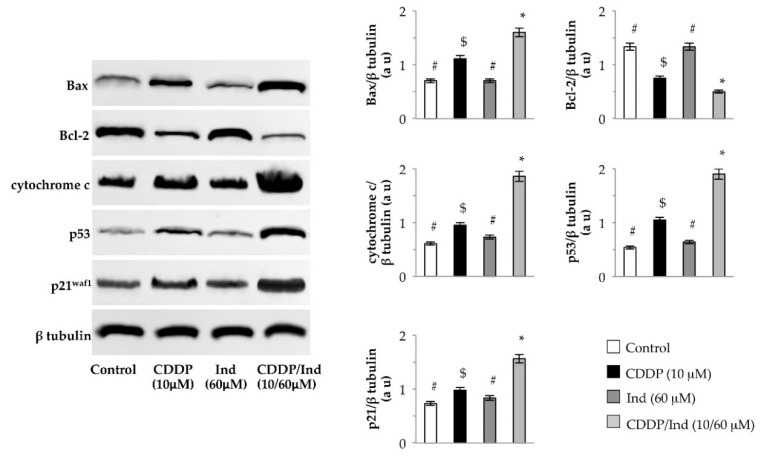
Effect of CDDP, Ind and CDPP/Ind on expression of apoptosis- and cell cycle-related proteins in HeLa cells. Cells were treated for 24 h as described in Methods. Representative images of immunoblotting analysis of Bax, Bcl-2, cytochrome c, p53 and p21waf1 levels and mean values ±SD of the densitometric analysis of four separate experiments. β tubulin was used as the internal control. au, arbitrary units. Bars without a common symbol are significantly different with *p* < 0.05 (one-way Anova associated with Tukey’s post hoc test).

Mitochondrial dysfunction governs the intrinsic apoptosis pathway. We used western immunoblotting to assess the levels of Bax, Bcl-2 and cytochrome c as markers of this process. When compared to untreated cells (control), treatment with Ind alone did not change the level of the examined proteins, while expression of Bax and cytochrome c were markedly increased as well as that of Bcl-2 decreased by CDDP (*p* < 0.05). Interestingly, combined treatment with CDDP/Ind significantly raised the levels of Bax and cytochome c with reduced Bcl-2 level when compared to the treatment with CDDP alone (Figure 7).

Bax and Bcl2 genes are regulated by p53 transcription factor [24]. In HeLa cells we found that CDDP upregulated p53 levels. A higher p53 expression (*p* < 0.05) was found in cells co-treated with CDDP and Ind (Figure 7). Among the transcriptional targets of p53, also p21^waf1^ plays important roles in regulating cell growth arrest and apoptosis [25]. Combined treatment with CDDP and Ind significantly increased the p21^waf1^ level relative to the treatment with CDDP alone (*p* < 0.05) (Figure 7).

### 3.6. Effect of CDDP and Ind Alone and in Combination on the Cell Redox Balance

**Figure 8:**
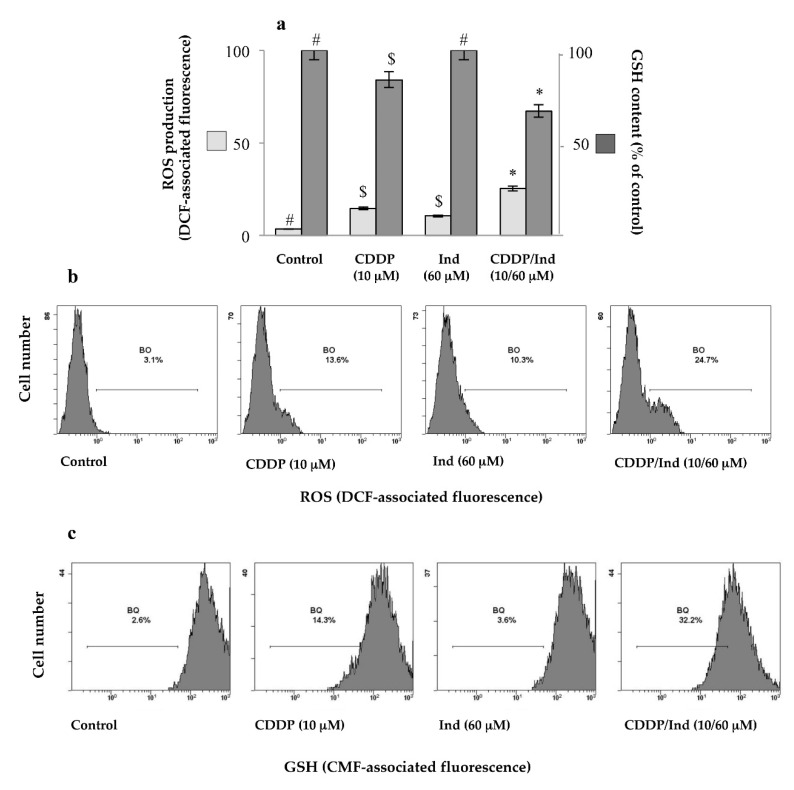
Cell redox unbalance induced in HeLa by CDDP, Ind and CDDP/Ind combination. ROS production and GSH levels were evaluated cytofluorimetrically after 24 h treatment by staining of the cells with DCFDA and CMFDA, respectively. compared to untreated cells (control). (**a**) Mean values ±SD of three separate experiments in duplicate. Within the same parameter, means without a common symbol are significantly different with *p* < 0.05 (one-way Anova associated with Tukey’s post hoc test); (**b**) Representative imagines of ROS production. (**c**) Representative imagines of intracellular GSH levels.

Oxidative stress is one of the most important mechanisms involved in CDDP toxicity. Intracellular ROS production and GSH levels in HeLa cells were measured by cytofluorimetric analysis using DCFDA or CMFDA as appropriate fluorescence probes, respectively. Treatment for 24 h of the cells with CDDP at 10 µM caused a significant increase of the ROS levels (+11.6 ± 1.6%) associated to a drop in GSH content (−18.9 ± 1.2%) in comparison to control cells, respectively (*p* < 0.05) (Figure 8). On the other hand Ind (60 µM) did not significantly vary the DCFDA-associated fluorescence neither changed GSH content compared to untreated cells. Interestingly, when the two drugs were simultaneously added, we measured an increase of ROS production (+22.3 ± 2.1%) and a reduction in GSH content (31.8 ± 1.1%), more remarkable than that caused by CDDP (Figure 8).

### 3.7. Pretreatment with NAC Prevents Apoptosis Induced by CDDP and Ind

**Figure 9:**
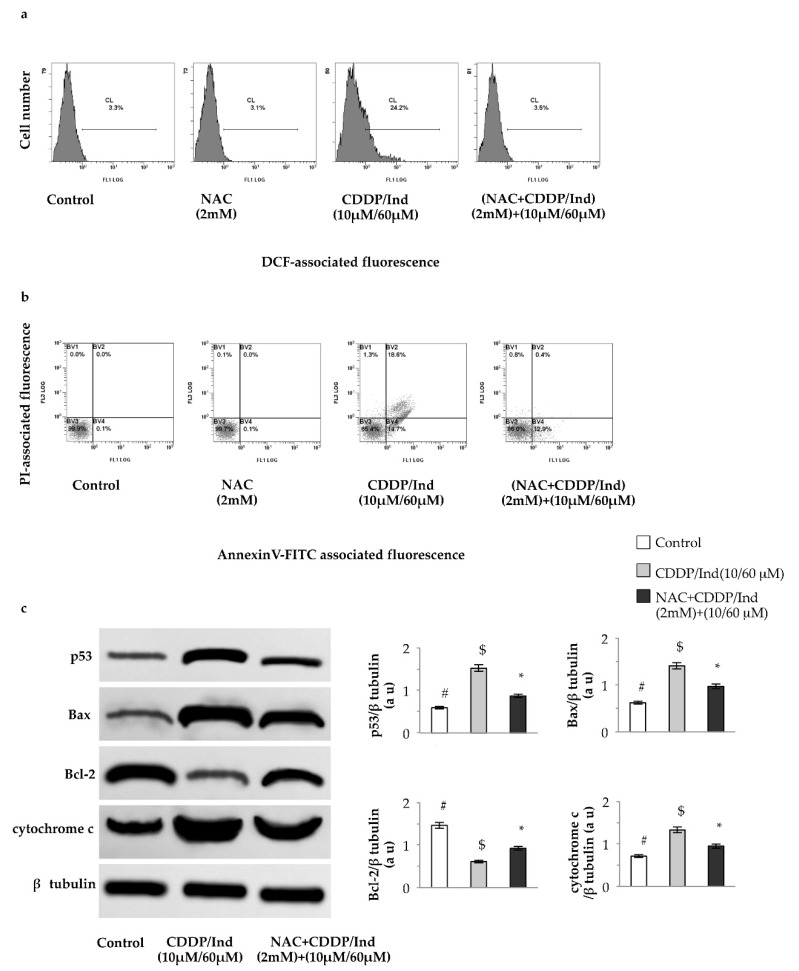
Pretreatment of HeLa cells with NAC prevents ROS-dependent apoptosis and modulates levels of apoptotic regulators induced by CDDP/Ind combination. Cells were treated for 24 h as described in Methods. (**a**) Intracellular ROS levels by flow cytometry using DCFA staining; (**b**) Apoptosis assay by flow cytometry using Annexin V-FITC /PI double staining; (**c**) Immunoblotting analysis of p53, Bax, Bcl-2 and cytochrome c levels with densitometric analysis. β-tubulin was used as the internal control. Representative images and mean values ±SD of three separate experiments. Means without a common symbol are significantly different with *p* < 0.05 (one-way Anova associated with Tukey’s post hoc test). au, arbitrary units.

To assess whether the elevated redox unbalance was related to the apoptosis induced by the combination of CDDP and Ind, HeLa cells were pretreated for 1 h with 2 mM NAC, a general ROS scavenger, followed by treatment with CDDP (10 µM) plus Ind (60 µM) for 24 h. The ROS levels and the proportion of apoptotic cells were determined by flow cytometric analysis. Treatment of the cells with NAC alone did not influence any of the measured parameters (Figure 9). On the contrary NAC prevented the ROS generation in HeLa cells treated with CDDP/Ind and the percentage of apoptotic cells, following the combined treatment, was reduced from 34.90 ± 2.19% in the NAC untreated group to 13.57 ± 0.19% in the NAC treated group (Figure 9); the difference was statistically significant (*p* < 0.05). Notably, pre-incubation of the cells with 2 mM NAC before adding of CDDP/Ind, significantly attenuated the p53, Bax and cytochome c expression levels measured in the absence of the antioxidant, while Bcl2 increased (Figure 9). However, NAC, except for the ROS production, failed to restore the control values.

### 3.8. Potentiation of the CDDP Cytotoxic Activity by Ind

**Figure 10:**
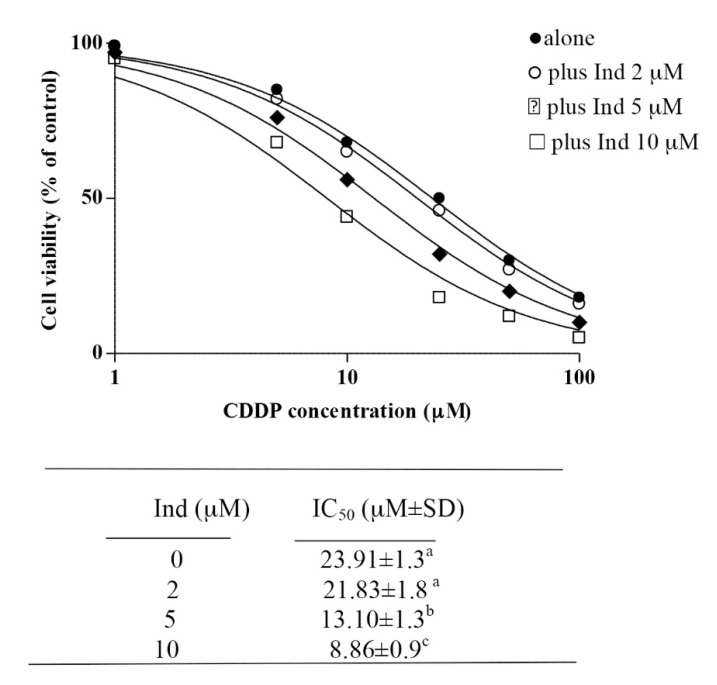
Cytotoxic effect of CDDP on HeLa cells in the absence or in the presence of not toxic Ind concentrations. Cells were treated for 24 h as described in Methods. IC_50_ values are the mean ±SD of three separate experiments in triplicate. Means with different superscript letters are significant *p* < 0.05 (one-way Anova associated with Tukey’s post hoc test).

We also investigated the potentiation of the anticancer effects of CDDP on Hela cells by low concentrations of Ind. Hela cells were incubated with Ind at 2, 5 or 10 µM in the absence or in the presence of variable concentrations of CDDP and cell viability was measured after 24 h of treatment. While Ind alone did not affect HeLa viability at any considered concentration (not shown), when the phytochemical was added in medium containing CDDP it caused an evident increase of the cytotoxic effect of the chemotherapeutic agent, such that higher the Ind amount was lower the cell vitality measured after treatment (Figure 10). When comparing the calculated IC_50_ values, co-treatment of Hela cells with 10 µM Ind lowered about three times the concentration of CDDP required for the reduction of the cell growth (Figure 10).

## 4. Discussion

Emerging evidence suggests that cancer-preventative phytochemicals may be combined with chemotherapeutic drugs for a more effective treatment of cancer. Dietary compounds such as genistein, curcumin, epigallocatechin-3-gallate, resveratrol and apigenin have all been recognized as tumor-preventing agents [26,27]. Their capacity of enhancing anticancer activity or minimizing the resistance of cancer cells to a number chemotherapeutics has been explored [7,9]. Ind, the characteristic pigment of cactus pear fruits, is a redox-active compound able to exert important poly-pharmacological effects included antiproliferative and tumor suppressor effects both in vivo and in vitro [15,16,17,28] and may have a chemopreventive potential. In this study, we show that Ind individually exhibits growth inhibitory activity on HeLa cervical cancer cells and when combined with CDDP cooperates in producing an enhanced anti-tumor effect against these cells. The chemosensitizing effect of Ind in combination with CDDP is causally associated with p53-dependent mitochondrial cell death pathway induced by oxidative stress.

Both Ind alone and CDDP alone were validated to have inhibitory effects on HeLa cells in a dose-dependent manner. Our data, obtained from the Combination Index analysis, demonstrated that combination regimens with Ind and CDDP produced significant synergistic anticancer effects (CI < 1) in a large range of concentrations. CDDP primarily exhibits its anticancer effects by inducing apoptosis in cancer cells [29]. Flow cytometry and fluorescence microscopy analysis showed that CDDP/Ind combined treatment caused a more significant apoptosis in HeLa cells than either CDDP or Ind treatment alone. As recent evidences suggest that the apoptosis induction efficacy of antitumor drugs gradually decreases due to chemoresistance [30,31], our results suggest that Ind may be a potential candidate for strengthening the apoptotic effect of low CDDP doses, then delaying the unwanted effect of chemoresistance of the drug in the cervical tumor.

A number of checkpoints that ensure the accuracy and integrity of the DNA replication control cell cycle progression. Cancer is associated with defects in the cell cycle checkpoints, which prevent the cycle from stopping in spite of adverse conditions. One of the anticancer mechanisms of CDDP is the activation of cell cycle checkpoints, which induce a transient S-phase arrest of cancer cells [29], as also shown in our study. However, the combination of Ind and CDDP caused a block of the cell cycle at G0/G1 phase, indicating that chemosensitization occurs during the execution of the G1 phase. The induction of p21^waf1^, known to silence the G1/S-promoting cyclin E/CDK2 kinase [25], detected in HeLa cells co-treated with CDDP/Ind, can account for the observed inhibition of the cell cycle transition with G0/G1 arrest. Other natural chemopreventive agents, such as myricetin and methyl eugenol in combination with CDDP have been reported to significantly induce an arrest of HeLa cells in the G0/G1 phase [10].

Mitochondria support and play a critical role in apoptosis and a fall of MMP, caused by an increment of Bax/Bcl2 ratio, is a crucial event in the intrinsic cell death pathway [32]. In the present study, the MMP of HeLa cells was significantly decreased in the CDDP/Ind group, compared with that in the CDDP-alone; in addition a stronger decrease in the antiapoptotic protein Bcl-2 with an increase in the proapoptotic proteins Bax were evidenced. These results demonstrated that combined treatment with CDDP/Ind can effectively induce apoptosis in HeLa cells through the mitochondrial apoptotic pathway.

p53 is a key protein for several fundamental cellular signaling pathways [33]. The most important of these pathways for p53′s tumor-suppressive role are the induction of apoptosis and cell cycle arrest [34]. p53 acts as a sequence-specific nuclear transcription factor that regulates transcription of genes controlling mitochondrial permeability, such as Bcl2 protein family, as well as the cell cycle progression, such as p21^waf1^ [35]. In our study, immunoblotting analysis revealed an increased accumulation of p53 in HeLa cells co-treated with CDDP and Ind with respect to cells treated with CDDP alone. These results indicate that Ind cooperatively amplified the CDDP cytotoxic effect through induction of p53 and p53 regulated proapoptotic and growth inhibitory gene expression. Similarly crocin, a naturally derived compound obtained from the stigma of *Crocus sativus* (saffron) and crocetin, the major constituent of saffron, have been reported to enhance the chemosensitivity of CDDP in lung and esophagus cancer cells, respectively, by upregulation of p53/p21^waf1^ and Bax levels with a concomitant decrease of Bcl-2 [36,37].

Cancer cells are usually under a higher oxidative stress than normal cells and an additional increment of the ROS generation can trigger cell death [38,39,40]. Main antineoplastic drugs that are currently used for cancer chemotherapy such as anthracyclines, taxanes and platinum-related compounds, including cisplatin, induce higher oxidant levels [41,42]. Accordingly, the combination of natural compounds with chemotherapics to increase the redox state to the condition of oxidative stress may be considered a successful adjuvant therapy against cancers. Ind is a redox active compound capable of both antioxidant and pro-oxidant effects, depending on its concentration [12,43]. In our study, although Ind alone at 60 µM was not capable of significantly modifying the redox status of HeLa cells in comparison with the control group, when combined with CDDP caused a net raise of ROS and a more marked decay of GSH content respect to CDDP alone group. Moreover, we found that pre-treatment with the ROS scavenger NAC blunted the CDDP plus Ind-induced PS externalization as well as the expression of mitochondrial apoptosis-related proteins. Collectively, our data demonstrates that the chemo sensitizing effect of Ind in combination with CDDP is causally associated with oxidative stress-mediated mitochondrial cell death pathway and that ROS act as an upstream signaling molecule to initiate p53-mediated apoptosis when HeLa cells were co-treated with CDDP and Ind. Considering these encouraging data, our future studies will be directed toward the analysis of the chemotherapeutic or chemosensitizer potential of Ind when used in combination with the most common anti-neoplastic agents in animal tumor models.

Dietary modification for patients receiving chemotherapy has been a regular practice to aid better digestion and adequate nutrition. Today, the intake of food as a dietary intervention to provide a variety of chemotherapy-sensitizing natural agents and overcome chemoresistance is a novel concept in oncology [44]. Ind is a highly bioavailable phytochemical. After a single ingestion of 500 g of cactus pear fruit pulp, it reaches in humans a maximum plasma concentration of about 7.0 µM at 3 h after eating the fruits [18]. We show that pre-treatment of Hela cells with Ind at low micromolar concentrations of nutritional relevance, significantly potentiates the cytotoxic action of CDDP, reducing its semi-maximal effective dose. Other studies in healthy men and women have provided a good indication of the potential of this fruit to positively affect the body’s redox balance, to decrease oxidative damage to lipids and improve antioxidant status and the response of the immune system [45,46]. Our data suggests that diet intervention with *Opuntia cactus pear* fruits in patients with cervical cancer undergoing chemotherapy might be considered with the aim to provide Ind as a chemosensitizer of the therapy.

## Figures and Tables

**Table 1:** Dose-response association of CDDP and Ind alone or in combination.

Compound	IC_50_	r	CI Values			DRI Values		
	(µM)		*f_a_*_0.25_	*f_a_*_0.5_	*f_a_*_0.75_	*f_a_*_0.25_	*f_a_*_0.5_	*f_a_*_0.75_
CDDP	23.91	0.99	0.71	0.66	0.76	3.72	2.97	2.30
Ind	149.55	0.93				2.25	3.06	4.16
CDDP/Ind	56.51	1.00						

DRI, dose reduction index; r, correlation coefficient.
